# Detection of apoptosis by [^18^F]ML-10 after cardiac ischemia–reperfusion injury in mice

**DOI:** 10.1007/s12149-022-01801-0

**Published:** 2022-10-28

**Authors:** Maximilian Fischer, Mathias J. Zacherl, Jessica Olivier, Simon Lindner, Steffen Massberg, Peter Bartenstein, Freba Grawe, Sibylle Ziegler, Matthias Brendel, Sebastian Lehner, Guido Boening, Andrei Todica

**Affiliations:** 1 Medizinische Klinik Und Poliklinik I, Klinikum Der Universität München, Ludwig-Maximilians-Universität, Marchioninistrasse 15, 81377 Munich, Germany; 2 DZHK (German Centre for Cardiovascular Research), Partner Site Munich Heart Alliance, 80802 Munich, Germany; 3 Department of Nuclear Medicine, University Hospital, LMU Munich, Marchioninistr. 15, 81377 Munich, Germany; 4 Department of Radiology, University Hospital, LMU Munich, Munich, Germany; 5 Die Radiologie, Munich, Germany

**Keywords:** Positron emission tomography, Autoradiography, [^18^F]ML-10, [^18^F]FDG, Apoptosis, Cardiac ischemia–reperfusion injury

## Abstract

**Objective:**

Myocardial infarction leads to ischemic heart disease and cell death, which is still a major obstacle in western society. In vivo imaging of apoptosis, a defined cascade of cell death, could identify myocardial tissue at risk.

**Methods:**

Using 2-(5-[^18^F]fluoropentyl)-2-methyl-malonic acid ([^18^F]ML-10) in autoradiography and positron emission tomography (PET) visualized apoptosis in a mouse model of transient ligation of the left anterior descending (LAD) artery. 2-deoxy-2-[18F]fluoro-D-glucose ([^18^F]FDG) PET imaging indicated the defect area. Terminal deoxynucleotidyl transferase dUTP nick end labeling (TUNEL) histology stain indicated cardiac apoptosis.

**Results:**

[^18^F]ML-10 uptake was evident in the ischemic area after transient LAD ligation in ex vivo autoradiography and in vivo PET imaging. Detection of [^18^F]ML-10 is in line with the defect visualized by [^18^F]FDG and the histological approach of TUNEL staining.

**Conclusion:**

The tracer [^18^F]ML-10 is suitable for detecting apoptosis after transient LAD ligation in mice.

## Background

Ischemic heart disease is still a burden in western society [1, 2].

Ruptured atherosclerotic plaques in coronary vessels result in cardiac ischemia and loss of viable tissue. Coronary bypass surgery and percutaneous coronary intervention (PCI) aim to re-establish the perfusion of ischemic cardiac tissue [3]. Translational animal models decipher ongoing cardiac processes after injury and aim to reduce the burden of ischemic heart disease. A better understanding could transfer novel diagnostic and therapeutic applications into the daily clinical scenario, thereby reducing the number of patients suffering from ischemic heart failure.

Plenty of reviews depict the intracellular mechanism of apoptosis, a process of controlled cell death [4–6]. In brief, the intrinsic and extrinsic pathways leading to apoptosis converge in the activation of executor enzymes, so-called caspases, and result in biochemical and morphological alteration of apoptosis (e.g., chromatin condensation, shrinkage, and breakdown of proteins and DNA [7, 8]). This process’s main feature is the externalization of phospholipid phosphatidylserine into the cell membrane [5].

Positron emission tomography (PET) could offer a feasible method for in vivo imaging myocardial damage. Imaging apoptosis induced by ischemic heart injury could implement novel diagnostics and monitor therapeutic interventions [9–11].

The small molecule probe, 2-(5-fluoropentyl)-2-methyl-malonic acid (ML-10) (206 Da), could display a promising approach in apoptosis imaging. ML-10 is incorporated and accumulated in apoptotic cells, not viable or necrotic cells, thereby discriminating those different pathologies visualized by in vivo imaging [12].

PET probes for imaging apoptosis such as Annexin-V have been extensively studied**.** Annexin-V helped in detecting apoptosis in myocardial infarction, atherosclerotic plaques, and in monitoring cancer therapies [13–16]. However, some limitations prevented the translation of Annexin-V into the clinic. Annexin-V binds to numerous phosphatidylserine head groups on cell surfaces, and is thereby limited by its specificity and its ability to discriminate apoptotic and necrotic cells, the slow clearance of non-targeted tissues, and its large protein structure (36 kDa) (reviewed in [10, 17]). Therefore, new strategies are needed for the development of imaging cell death.

[^18^F]ML-10 visualized apoptotic cancer cells [18] and evaluated the effect of radiation therapy on brain cancer and metastasis [19, 20].

In a murine stroke model induced by ligation of the arteria cerebri, the molecular probe [^18^F]ML-10 was feasible to detect ischemia, while there was no accumulation in non-ischemic brains [21]. Regarding cardiovascular disease models, [^18^F]ML-10 accumulation detected apoptotic cells within atherosclerosis-like lesions in rabbits [22]. Injured aortas were further evaluated by ex vivo autoradiography and histology that identified the [^18^F]ML-10 accumulation. Two surgical myocardial infarction models are established to mimic ischemic cardiac injury [23, 24]. The permanent ligation of the LAD artery without reperfusion results in a significant cardiac defect. On the other hand, the transient LAD ligation enables the translation of the early recanalization into an animal model, resulting in ischemia–reperfusion injury (reviewed in [25]). Recently, our group showed that [18F]ML-10 could be used to detect apoptosis after permanent ligation of the LAD in mice illustrating an untreated myocardial infarction [26]. Here, we show data of [^18^F]ML-10 in the ischemia–reperfusion injury, resembling the model of re-established perfusion in myocardial infarction, which represents an important model of myocardial infarction besides permanent LAD ligation.

This study aimed to assess [^18^F]ML-10 in mice after transient LAD ligation inducing cardiac ischemia–reperfusion by autoradiography, cardiac PET imaging, and histology.

## Materials and methods

### Animals

The ischemia–reperfusion injury was induced in 10-week-old C57BL/6 N mice by transient surgical ligation of the LAD, as described previously [4, 5, 27]. Male C57BL/6 N mice were purchased from Charles River (Germany). In total, 52 mice were analyzed in this study: for autoradiography three mice were sacrificed for each timepoint (2, 4, 6, 24, and 48 h; in total 15 mice), for in vivo PET imaging and subsequent TUNEL histology, in total 32 mice were analyzed at the respective timepoints (2 h: 7 mice, 4 h: 7 mice, 6 h: 7 mice, 24 h: 7 mice (data for one mouse at this timepoint failed in the reconstruction process), 48 h: 4 mice), and in total 5 sham control mice.

Animal care and experiments were performed according to the current Guideline for the Care and Use of Laboratory Animals (US National Institutes of Health).

In brief, after inducing anaesthesia by intraperitoneal injection of medetomidine 0.5 mg/kg, midazolam 5.0 mg/kg, and fentanyl 0.05 mg/kg. Mice were placed supine with paws taped to the operation table. An incision along the midline cervical was made to reflect the muscles overlying the trachea, thus visualising the endotracheal tube. After surgical preparation, the mice were intubated with a 19 gauge tube and ventilated at a volume of 0.15 ml and a frequency of 110/min (Mini Vent T845, Hugo Sachs Elektronik, Hegstetten). After tube placement, the cervical skin was sutured (5-0 Ethibond). The mice were positioned on a heating mat, and a rectal probe closely monitored their body temperature. The third intercostal space was prepared for thoracotomy. The LAD artery was ligated by an 8-0 prolene suture and resulted in ischemia of the left ventricle. The diminished blood flow distal to the ligation site was used as intra-operative criteria for successful ligation. To enable reperfusion injury, the suture was removed after 30 min. Sham-operated mice received the same treatment except for the transient LAD ligation. 6-0 Ethibond suture closed the chest wall. Post-analgetic was applied (Buprenovent 0.3 mg/ml) subcutaneously. Anaesthesia was reversed by 2.5 mg/kg atipamezole and 0.5 mg/kg flumazenil. The animal was removed from the respirator, the endotracheal tube was withdrawn, and the animal was kept warm on the heating mat till transferred into cages after recovery from anaesthesia. After surgery, [^18^F]ML-10 was injected at the indicated time points (2, 4, 6, 24, and 48 h). The harvesting of the hearts for autoradiography and PET emission acquisition was performed 135 min post-injection to enable valid uptake comparison. Immediately after PET imaging, the hearts were excised and fixed for further histological analysis.

Study protocols complied with the institution’s guidelines and were approved by the Government’s animal ethics committee (ROB-55.2Vet-2532.Vet_02-15-241).

### Radiolabeling procedure of ML-10

Radiolabeling of ML-10 was performed as described previously [26].

### Autoradiography

Intravenous injection of [^18^F]ML-10 (~ 16 Mbq) was performed 135 min before sacrificing. The heart was perfused with 0.9% sodium chloride solution and stored as cryosections at − 80 °C. Cryosections of the heart of 30 µm were used. The heart sections were put into the imaging plate, and after 12 h, the heart slices were analyzed using a HD-CR 35 NDT image plate scanner. The images were quantified using advanced image data analyzer (AIDA). The circular region of interest (ROI) was applied to each heart section for the infarct and remote area at the mid-ventricular section of the heart. The non-infarct area (remote area) above the surgical LAD ligation was used as background estimation.

### In vivo cardiac PET imaging

[^18^F]PET scans were performed according to the indicated time points using an Inveon small-animal PET scanner (Preclinical Solutions, Siemens Healthcare Molecular Imaging, Knoxville, TN, USA) as described previously [27, 28].

The animals had free access to food and water until before the scan, as described previously [27–30]. Anaesthesia was induced (2.5%) and maintained (1.5%) with isoflurane delivered in pure oxygen at a rate of 1.5 L/min after intubation and mechanical ventilation. A heating pad and rectal thermometer were used to control the body temperature. First, the PET imaging for [^18^F]ML-10 was performed, followed by [^18^F]FDG imaging. Approximately 16 MBq of [^18^F]ML-10 was injected in a volume of ~ 150 µl into a tail vein. The catheter was then flushed with 50 µl of saline solution.

The isoflurane narcosis was interrupted for the time of [^18^F]ML-10 tracer uptake.

The animals were re-anaesthetized using isoflurane and placed in a prone position in the PET scanner. A three-dimensional PET recording was obtained in list mode for 30 min after [^18^F]ML-10 injection. A 7-min transmission scan was performed with a rotating Co-57 source, followed by 30-min emission for attenuation and scatter correction.

After the [^18^F]ML-10 scan, the [^18^F]FDG was performed. Approximately 20 MBq of [^18^F]FDG in a volume of ~ 100 µl was injected into the tail vein. The catheter was again flushed with 50 µl of saline solution. After 13 min, the transmission was again assessed for 7 min, followed by the assessment of emission for 20 min. After the [^18^F]FDG PET scan, mice were sacrificed. Reconstruction for [^18^F]FDG and [^18^F]ML-10 was performed using a MAP OSEM 3D algorithm in a 256 × 256 × 159 matrix and dimension of 0.39 × 0.39 × 0.8 mm^3^ using the Inveon Acquisition Workplace (Siemens Medical Solutions, Knoxville, TN, USA). Data were reconstructed as a static image, normalized, and corrected for randoms, dead time, decay, attenuation, and scatter.

### PET image analysis

Analysis of PET images was performed by the Inveon Research Workplace (Siemens Medical Solutions) described previously [31, 32].

Inveon Research Workplace was used for cutting, fusion, and assessing [^18^F]ML-10 and [^18^F]FDG PET images. A circular volume of interest (VOI) was used to evaluate the infarct area and remote area (basal interventricular septum), which was confirmed by the [^18^F]FDG-directed localization. The volume of interest (VOI) was verified in axial, sagittal, and coronal projections. The maximum injected dose per gram (% ID/g)_max_ was determined as the quotient of maximum uptake per ROI (Bq/mL) to injected dose/activity in Bq multiplied by 100. Tissue density was set as 1 g/ml.

### TUNEL staining

According to the manufacturer's protocol, apoptotic cells were stained using ApopTag^®^ Peroxidase In Situ Apoptosis Detection Kit S7100 (Millipore), and the Axio Version SE64 (Version 4.9) software was used for evaluation. Hearts were excised after the PET scans and fixed in 4% phosphate-buffered formalin. 4-µm thick sections were embedded in paraffin.

### Statistical analysis

Statistical analysis was performed with GraphPad Prism (Version 9, GraphPad Software). One-way ANOVA analysis with Tukey’s multiple comparisons was applied. The Wilcoxon signed-rank or the Mann–Whitney *U* test was applied for groups without normal distribution. Two-tailed Pearson correlation was used to perform the correlation analysis. All results depict the mean with the standard error of the mean. A *P* value of 0.05 was used for significance.

## Results

### [^18^F]ML-10 uptake in the heart after transient LAD ligation using autoradiography

**Fig. 1:**
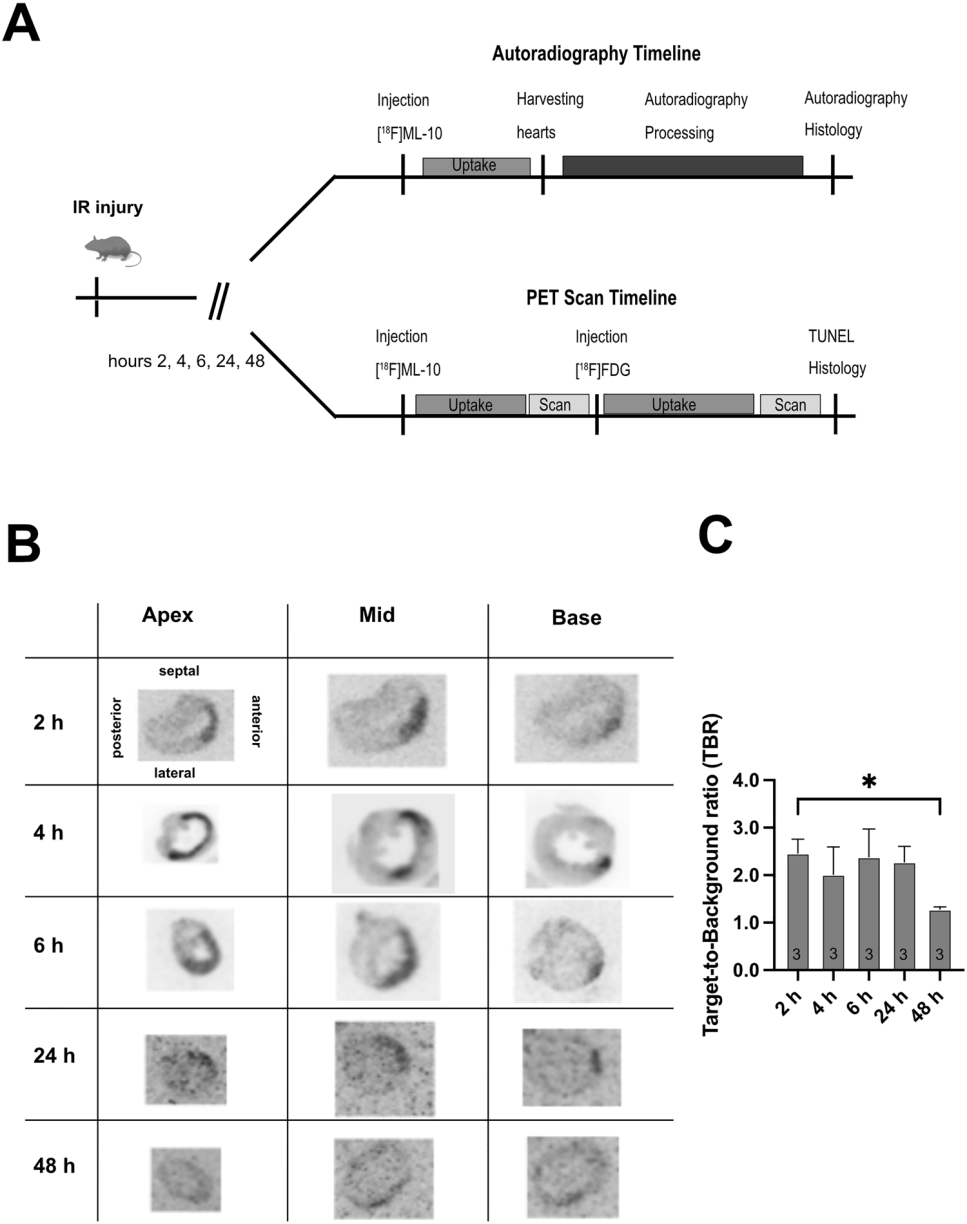
Autoradiography of [^18^F]ML-10 after transient LAD ligation. **A** Schematic study design illustrating the induction of ischemia–reperfusion (IR) injury by transient LAD ligation, application of tracers [^18^F]ML-10 and [^18^F]FDG, autoradiography, PET scan, and TUNEL histology at the indicated timeline. **B** [^18^F]ML-10 autoradiography after transient LAD ligation according to the timeline. Hearts are illustrated at different sections (apex, mid-ventricular, base). **C** Quantification of the target-to-background ratio (TBR) of [^18^F]ML-10. *N* = 3. Data represent mean ± SEM. *p < 0.05, **p < 0.01, ***p < 0.001

The uptake of [^18^F]ML-10 after transient LAD ligation was assessed by autoradiography at different times (2, 4, 6, 24, and 48 h). Figure 1A depicts the experimental timeline for autoradiography and PET imaging.

The hearts were excised at the indicated time after transient LAD ligation, and autoradiography of the harvested hearts was performed by evaluating the target-to-background ratio (TBR) (Fig. 1B).

After transient LAD ligation, [^18^F]ML-10 accumulated early with a peak at 2–6 h and then declined (IR 2 vs. 48 h, *P* = 0.016, Fig. 1C**)**.

Our observations indicate that the cardiac [^18^F]ML-10 accumulates after transient LAD ligation and declines at 48 h.

### Small-animal PET imaging of [^18^F]FDG and [^18^F]ML-10

**Fig. 2:**
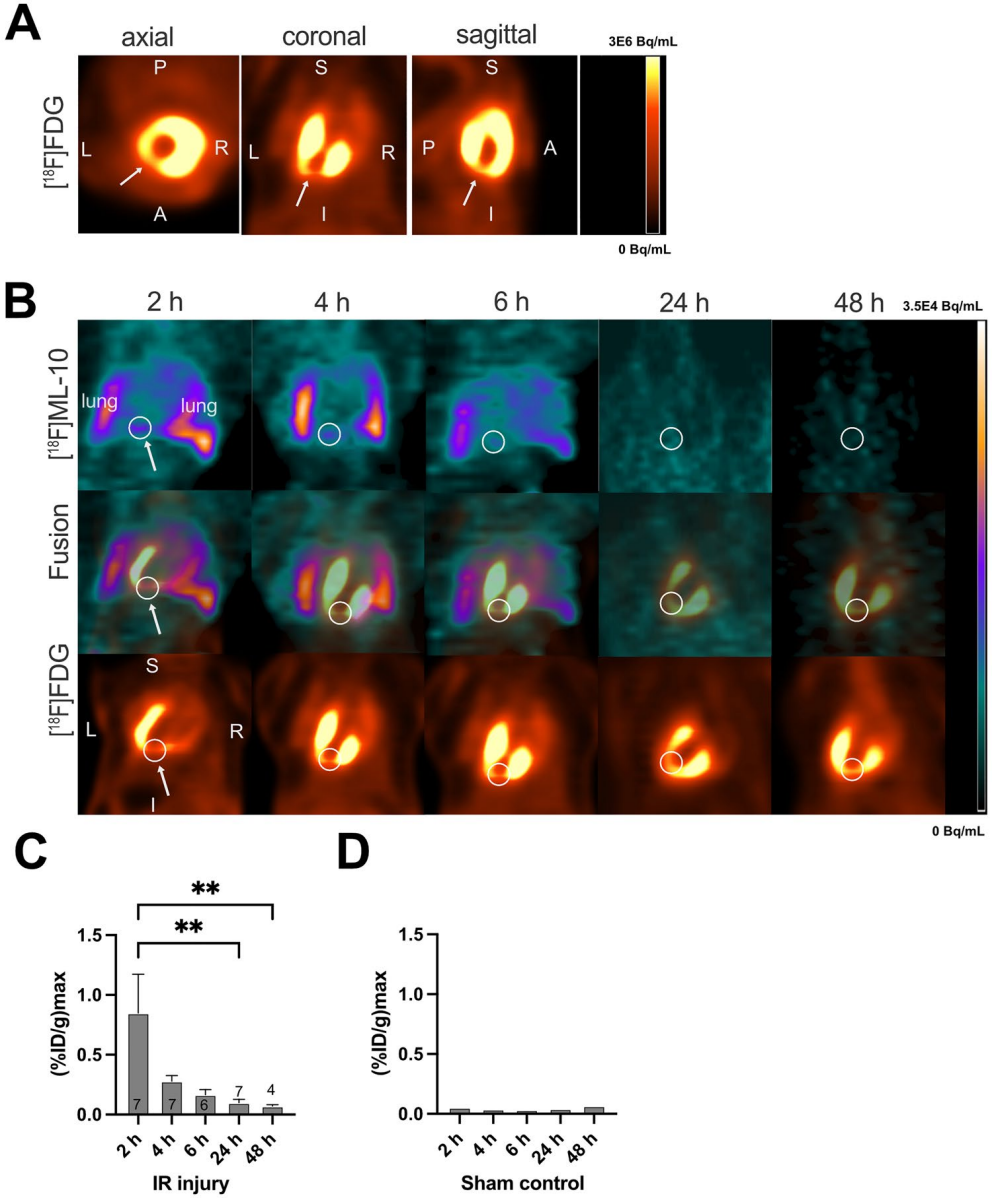
[^18^F]FDG and [^18^F]ML-10 PET imaging after transient LAD ligation. **A** Representative [^18^F]FDG image illustrating the cardiac injury after transient LAD ligation. Arrows indicate the infarct area of the left ventricle. *R* right, *L* left, *P* posterior, *A* anterior, *S* superior, *I* inferior. Colour scale: Volcano. **B** Representative fusion images of [^18^F]FDG and[^18^F]ML-10 in coronal orientation. White circles and arrows indicate infarct area detected by diminished [^18^F]FDG. PET images are illustrated for each time point. *R* right, *L* left, *S* superior, *I* inferior. Lung indicated in white letters. Colour scale: ocean and volcano. **C** [^18^F]ML-10 quantification (%ID/g max) after different times *N* = 4–7. Data represent mean ± SEM. *p < 0.05, **p < 0.01, ***p < 0.001. **D** [^18^F]ML-10 quantification (%ID/g max) in sham-operated control mice. One mouse was analyzed at each time point

We further assessed the feasibility of in vivo monitoring of the cardiac [^18^F]ML-10 accumulation after transient LAD ligation. In vivo imaging of [^18^F]FDG was used to localize the defect area and validate the correct region of interest for [^18^F]ML-10 uptake. Using [^18^F]FDG PET imaging, the ischemic area indicated by the low [^18^F]FDG uptake could be adequately identified (Fig. 2A). In vivo [^18^F]ML-10 PET imaging was performed at the indicated time course, and the two PET images were fused to evaluate the [^18^F]ML-10 uptake in the ischemic area (Fig. 2B). As described previously [26, 33], [^18^F]ML-10 also accumulated after thoracotomy and in the lung. Nevertheless, we could detect a peak maximum of the cardiac uptake of [^18^F]ML-10 after 2 h in the infarct area and a dynamic decline the following hours (IR 2 vs. 24 h, *P* = 0.007, 2 vs. 48 h, *P* = 0.009, Fig. 2C). No relevant uptake could be detected in sham-operated mice (Fig. 2D).

### Detection of apoptosis by TUNEL staining after transient LAD ligation

**Fig. 3:**
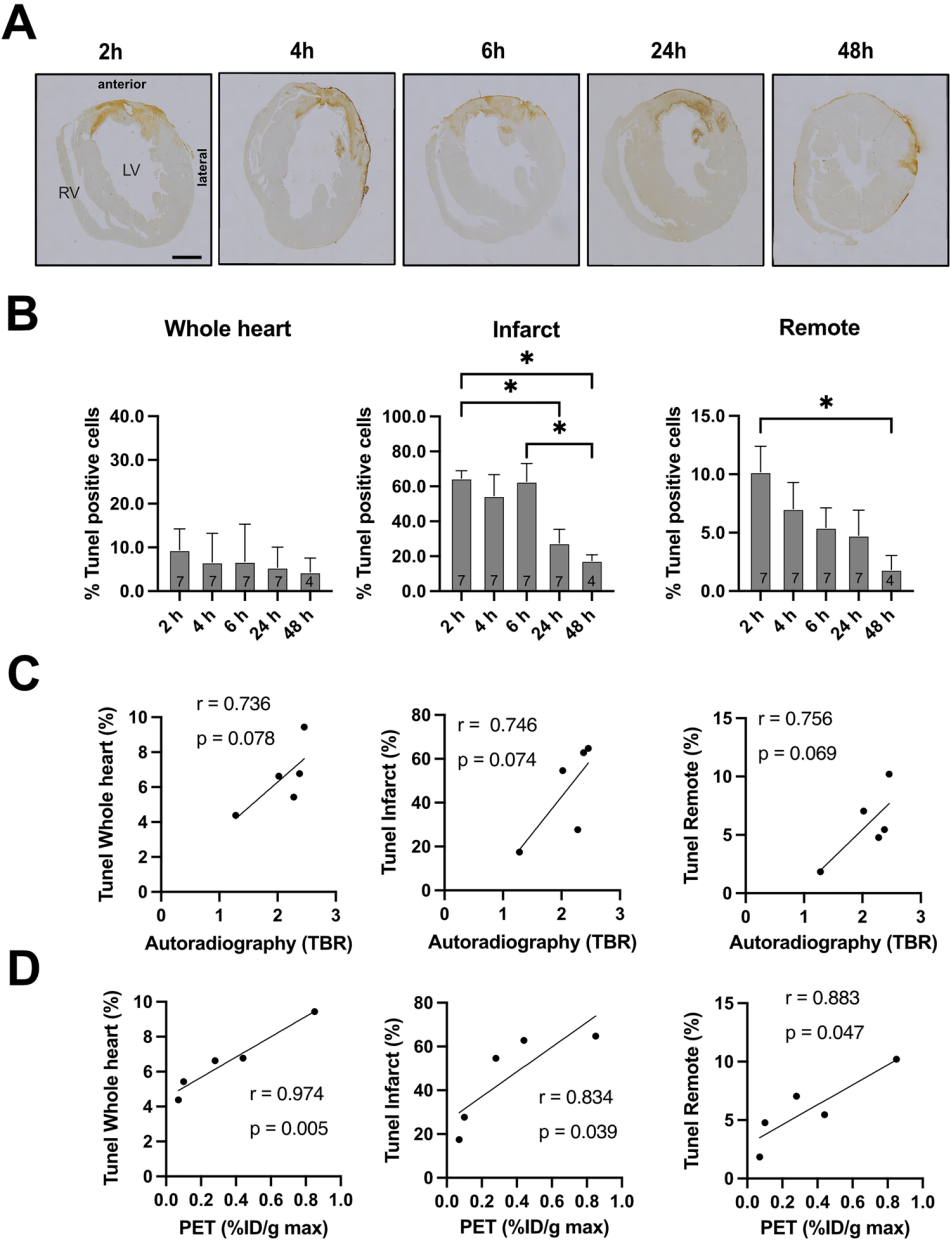
Histological evaluation by TUNEL staining. **A** Representative TUNEL staining after transient LAD ligation for each time point. Bar equals 1 mm. Data represent mean ± SEM. *p < 0.05, **p < 0.01, ***p < 0.001. **B** Quantification of TUNEL-positive cells in the whole heart, infarct area, and remote area. *N* = 4–7. **C** Correlation analysis of autoradiography and TUNEL staining. **D** Correlation analysis of [^18^F]ML-10 PET and TUNEL staining

Apoptosis was analyzed by TUNEL staining after transient LAD ligation (Fig. 3A).

The extent of apoptosis was analysed for the whole heart, infarct, and remote areas. Regarding the whole heart after transient LAD ligation, we detected ~ 9–10% of TUNEL-positive cells.

Most apoptotic cells were in the infarct area (Fig. 3B). After 24 h, we observed less apoptotic cells (IR 2 vs. 24 h, *P* = 0.034**,** IR 2 vs. 48 h, *P* = 0.019, IR 6 vs. 48 h, *P* = 0.072)**.** In the remote area, we observed TUNEL-positive cells 2 h after the operation that decreased over time (IR 2 vs. 48 h, *P* = 0.024).

Next, we performed correlation analysis to compare the ex vivo autoradiography and in vivo PET imaging with histology. We found a suggestive positive correlation comparing autoradiography towards the apoptosis regarding the whole heart (*r* = 0.736), the infarct area (*r* = 0.746), and the remote area (*r* = 0.756), which did not meet the significance criteria (Fig. 3C).

However, in vivo [^18^F]ML-10 PET imaging showed a strong and significant correlation for the whole heart (*r* = 0.974), the infarct area (*r* = 0.834), and the remote area (*r* = 0.883) (Fig. 3D).

## Discussion

This study is the first to assess the characteristics of [^18^F]ML-10 in a mouse model of cardiac ischemia–reperfusion injury. Using autoradiography, small-animal PET imaging, and histology by TUNEL stain, we assessed the feasibility of [^18^F]ML-10 after cardiac ischemia–reperfusion injury.

Previous studies evaluated the feasibility of [^18^F]ML-10 for apoptosis detection in rats after MI [33]. The accumulation of [^18^F]ML-10 was detected early on the first day till the third day after permanent LAD ligation. No uptake was detected at later stages, such as days 5 and 7 post-operation. Detecting apoptosis in human cardiac tissue after myocardial infarction is applicable relatively early after injury (ranging from 2 to 3 h) [34].

Previously, we could show that [18F]ML-10 accumulates in the defect area after permanent LAD ligation [26]. However, the model of transient LAD ligation inducing the ischemia–reperfusion injury better translated the clinical scenario of cardiac reperfusion of an occluded artery (e.g., by percutaneous coronary intervention or bypass surgery), and therefore preserved ejection fraction into pre-clinical research. While the permanent LAD ligation model is, on the other hand, a pivotal model for myocardial infarcts that are not treated in time, and therefore show profound scarring, left ventricular dilatation and heart failure with reduced ejection fraction [35]. Consequently, both models of myocardial infarction are distinct and of pivotal importance in basic cardiac research. The reperfusion of ischemic cardiac tissue could influence the results due to tracer availability in the ischemic area.”

Consequently, imaging early apoptosis after ischemic cardiac injury could provide a promising approach. We could detect a valid uptake of [^18^F]ML-10 in the autoradiography, as indicated by the increased TBR. Our data show a dynamic process since the TBR normalized 48 h after cardiac injury. In the next step, [^18^F]FDG PET imaging was utilized to localize the defect area in vivo after myocardial injury, as described previously [36]. We identified the infarct area by diminished [^18^F]FDG, and could therefore estimate the [^18^F]ML-10 uptake distal to the transient LAD ligation. Importantly, we could not detect a relevant uptake of [^18^F]ML-10 in sham-operated control mice (see Fig. 2D).

Evaluation of the %ID/g max showed localized uptake and a steady decline in [^18^F]ML-10 after transient LAD ligation, which is in line with the decrease in TBR after 48 h. [^18^F]ML-10 PET imaging estimated the %ID/g max in the infarct area determined by the diminished [^18^F]FDG uptake.

In our experiments, we observed a different course of [^18^F]ML-10 in autoradiography compared to in vivo PET imaging at the early time points after cardiac injury. This observation could be due to a technical limitation represented by comparing in vivo and ex vivo analyses. While [^18^F]FDG was used to localize the PET imaging analysis in vivo, the autoradiography uses the TBR, which also considers the remote signal, which could affect the ratio itself. Ma et al. [33] also observed uptake in the remote area; however, they did not provide the autoradiography data, including the myocardial target-to-background ratio. Cohen et al. described that ML-10 indicates apoptosis [12]. The TUNEL-positive cells which could be detected in the remote myocardium after infarction [37] might explain the autoradiography data.

A limitation of this study is that [^18^F]ML-10 uptake was not limited to cardiac injury. The previous publication on rat myocardial infarct also observed the accumulation in the chest wound after thoracotomy [33]. The murine infarct model's limitations are oropharyngeal intubation, mechanical ventilation, and thoracotomy. In our experiments, we observed a non-specific [^18^F]ML-10 uptake reduction over time. While the mice were intubated and mechanically ventilated during surgery, the in vivo PET imaging for 48 h was performed by isoflurane mask narcosis and spontaneously breathing mice. This could explain the apparent reduction in lung uptake. On the other hand, the cardiac [^18^F]FDG signal, further enhanced by isoflurane narcosis, remained stable [36]. As expected**,** a decent [^18^F]FDG background signal was observed. Since the cardiac [^18^F]FDG uptake was enhanced by isoflurane narcosis, we did not further evaluate the [^18^F]FDG activity in the infarct area. Our experimental design of using different experimental groups, instead of longitudinal PET scans, avoided the potential bias of overlapping tracer activity. Using ketamine/xylazine narcosis, which is not influencing the cardiac [^18^F]FDG uptake after ischemia [38], would be the accurate strategy to evaluate the overlapping activities.

If this observation can be detected and better discriminated in larger animals or patients, as shown in rats, it remains to be elucidated in future in vivo investigations.

Furthermore, we assessed the cardiac apoptosis histologically by TUNEL staining, which is the most feasible approach to assess cardiac cell apoptosis [39]. It should be considered that TUNEL staining is used to detect DNA strand breaks, while membrane potential and acidification alterations cannot be detected. Therefore, the TUNEL method is limited by detecting both apoptosis and non-specific DNA degradation [39, 40]. Before using the TUNEL staining in our experimental setup, we also evaluated the staining for active caspase-3. We did not detect a signal of active caspase-3 in these early time frames (data not shown). According to the literature, caspase-3 staining is feasible after 24–72 h [41]. Thus, active caspase-3 staining would evaluate the myocardial ischemia at later stages than in our experiments. After transient LAD ligation, TUNEL-positive cells were evident in the infarct and remote area. The reperfusion effect presumably explained by the TUNEL-positive cells decreased in the infarct and remote areas. Literature suggests that reperfusion leads to an enhancement of apoptosis (reviewed in [42]). It is common sense that reperfusion restores the energy required to complete apoptosis and can accelerate the process [40, 43, 44]. Our correlation analysis indicates that the [^18^F]ML-10 uptake correlates with the number of TUNEL-positive cells by proper localization. A limitation of this work and the correlation analysis is that only the hearts after PET imaging underwent further histological analysis by TUNEL stain and not the ones after autoradiography. This could potentially explain that the positive trend in the correlations of autoradiography and TUNEL does not reach statistical significance. At the same time, there is a statistically significant correlation between PET and TUNEL staining. Another explanation for the positive trends that do not reach significance is further indicative that apoptosis is not being measured exclusive of other adjacent processes that occur during injury.

ML-10 was primarily described for selective incorporation into apoptotic cells but not into viable or necrotic cells due to specific membrane potential and acidification. The incorporation of ML-10 is ceased upon membrane disruption, thereby distinguishing apoptosis from necrosis [12]. This underlines the temporal dynamics of [^18^F]ML-10 after an ischemic heart injury and the decline over time.

This study displays the characteristic of [^18^F]ML-10 after transient LAD ligation but also bears several limitations. The surgical procedure led to a severe injury to the chest, and thus the detection of cardiac [^18^F]ML-10 PET imaging could have interfered.

Other publications underline this limitation, which was also observed in rat myocardial infarction experiments [33]. Substrate availability by blood flow, hormonal status, blood glucose levels, and insulin sensitivity could potentially interfere with the cardiac [^18^F]FDG uptake [45, 46]. Ma et al. showed that using pentobarbital in rats enables accurate infarct localization. In this work, mice under isoflurane narcosis were solely used to localize the defect area by reduced [^18^F]FDG uptake. Of note, we cannot fully exclude differences in rats versus mice regarding the FDG uptake pattern in the heart, especially using different narcotic agents. Importantly, our experimental strategy performed the injection and imaging of [^18^F]ML-10 first. Thus, there is no influence induced by the latter cardiac [^18^F]FDG uptake. In parallel, the search for a robust nuclear apoptosis tracer, the ongoing research on cardiac hypoxia (reviewed in [47]), or hypoxia identifying [^18^F]fluoromisonidazole (FMISO) [48] or the evaluation of the oxidative metabolism by [^11^C]acetate [49, 50] could display other suitable strategies.

## Conclusion

This mouse study indicates that [^18^F]ML-10 is a novel tracer for cardiac imaging injury after transient LAD ligation in mice. We confirmed the uptake by a multimodal approach, including autoradiography, in vivo small-animal PET imaging, and TUNEL staining. Our results underline that [^18^F]ML-10 could provide a novel approach for detecting cardiac injury in an ischemia–reperfusion model. Further investigation in larger animals such as pigs using endovascular interventions, should provide more insight into the applicability of [^18^F]ML-10 and better clarify the translational potential.

## Data Availability

The authors confirm that the data supporting the findings of this study are available within the article and/or its supplementary materials.

## Abbreviations

[^18^F]FDG: 2-Deoxy-2-[18F]fluoro-D-glucose
[^18^F]ML-10: 2-(5-[^18^F]fluoropentyl)-2-methyl-malonic acid
ID/g max: Maximum injected dose per gram
LAD: Left anterior descending artery
IR: Ischemia–reperfusion
PET: Positron emission tomography
ROI: Region of interest
TBR: Target-to-background ratio
TUNEL: Terminal deoxynucleotidyl transferase dUTP nick end labeling
VOI: Volume of interest
